# Evaluation of Biochemical, Hematological and Parasitological Parameters of Protein-Deficient Hamsters Infected with *Ancylostoma ceylanicum*

**DOI:** 10.1371/journal.pntd.0003184

**Published:** 2014-09-25

**Authors:** Carina P. Pacanaro, Sílvia R. Dias, Luciana R. Serafim, Mariana P. Costa, Edenil Aguilar, Paulo R. Paes, Jacqueline I. Alvarez-Leite, Élida M. Rabelo

**Affiliations:** 1 Departamento de Parasitologia, Instituto de Ciências Biológicas, Universidade Federal de Minas Gerais, Belo Horizonte, Minas Gerais, Brazil; 2 Departamento de Bioquímica e Imunologia, Instituto de Ciências Biológicas, Universidade Federal de Minas Gerais, Belo Horizonte, Minas Gerais, Brazil; 3 Faculdade Estácio de Sá de Juiz de Fora, Juiz de Fora, Minas Gerais, Brazil; 4 Laboratório de Patologia Clínica, Escola de Veterinária, Universidade Federal de Minas Gerais, Belo Horizonte, Minas Gerais, Brazil; Baylor College of Medicine, Texas Children's Hospital, United States of America

## Abstract

**Background:**

Hookworms infect millions of people worldwide and can cause severe clinical symptoms in their hosts. Prospective cohort studies in Brazil show high rates of hookworm reinfection in malnourished children compared to well-nourished children, despite previous treatment. Additionally, soil-transmitted helminth (STH) infections can worsen the nutritional status of affected populations. Therefore, this study aims to clarify the effects of host malnutrition during *Ancylostoma ceylanicum* infection and how this infection affects host physiological parameters using a hamster model.

**Methodology/Principal Findings:**

Hamsters were divided into four experimental groups: normal diet or low-protein diet (also referred to as “malnourished”) and *A. ceylanicum* infection or no infection. More severe pathogenesis was observed in the infected malnourished group, as demonstrated by significant decreases in the hemoglobin concentration, erythrocyte number and packed-cell volume compared to the non-infected malnourished group. Greater numbers of adult parasites and eggs were observed in the malnourished group compared to the control group; however, the oviposition rate was lower in the malnourished group. In general, greater values of total lipids were observed in malnourished animals compared to control animals, including lipids excreted in the stool.

**Conclusions:**

In this work, we have demonstrated that animals fed an isocaloric low-protein diet presented more severe pathogenesis when infected with *A. ceylanicum*. The increased lipid concentration in the liver and blood is related to the conversion of the excess carbohydrate into fatty acids that increase the concentration of triglycerides in general. Triglycerides were excreted in the feces, indicating that infection associated with malnutrition caused a greater loss of these molecules for this group of animals and confirming the hypothesis that both nutrition and infection are responsible for the malabsorption syndrome. Taken together, the results found in this work confirm the hypothesis that the nutritional condition of the host greatly influences the course of the infection.

## Author Summary

Hookworm's infection is a health problem that affects areas of poverty worldwide. Nutritional diseases, including dietary protein deficiency are also prevalent in those areas. It is well known that nutritional status can influence parasite infection, reducing host responses to infection. However, the influence of nutritional status and Hookworm's infection is less explored. Our goal was to study the consequences of coexistence of low-protein malnutrition and Hookworm's infection in hamsters that are good models of this infection. For that, we established a model of protein malnutrition by feeding hamsters on standard diet or isocaloric, low-protein diet for 50 days. Hamsters fed on those diets were infected with hookworm's larvae for assessment of biochemical and parasitological parameters compared to non-infected controls. Our results confirm that malnutrition was installed, as suggested by low antibody response and by increased fecal lipid excretion in malnourished group. Regarding Hookworm's infection, animals fed low-protein diet presented a more severe infection, with higher number of worms recovered in intestine. Nonetheless, egg production was reduced in malnourished hamsters, suggesting that protein restriction was determinant on egg formation. These results confirm the hypothesis that host nutritional status greatly influences the course of Hookworm's infection.

## Introduction

Among geohelminths of zoonotic significance, hookworms continue to be a major public-health problem, with millions of people infected in underdeveloped or developing countries [1]. Hookworms are hematophagous intestinal parasites and are a major cause of iron-deficiency anemia and malnutrition in children [2]–[7]. Adult worms cause intestinal bleeding, resulting in blood in the stool in amounts proportional to the parasite load in the intestine [7].

Malnutrition and intestinal parasites are co-prevalent in many areas, and these parasites are particularly prevalent in children, who also experience the most pathology [8]. The interaction between parasitism and nutrition has two interrelated facets: the influence of parasitism on host metabolism and nutrient absorption both exacerbates and affects the development of the parasite population in the host [9].

Approximately 826 million people worldwide are malnourished, including 792 million people in developing countries and 34 million in developed countries [10]. According to a WHO report analyzing child mortality in 2012, approximately 45 percent of under-five deaths were attributable to undernutrition globally [11]. There are two forms of protein-energy malnutrition (PEM). Marasmus is the most common form of severe energy malnutrition that affects children, and it primarily occurs because of the scarcity of food in areas of extreme poverty. It is known as dry malnutrition because affected children lose as much as 60% of their body weight. Kwashiorkor occurs due to very low quantities of protein and excess carbohydrates in the diet, leading to ongoing insulin secretion in the body. Insulin spares muscle protein but causes loss of protein from the liver. Thus, the production of plasma albumin and low-density lipoprotein are reduced, causing edema and lipid accumulation in the liver [12]. Kwashiorkor usually occurs in children who stopped breastfeeding because the mother had given birth to a new baby, following which the elder child was fed a diet very poor in protein, such as porridge made of corn starch, cassava or banana. It is classified as wet malnutrition because the child is at an appropriate weight, mainly due to swelling in the abdomen resulting from a reduction in plasma protein and lipid accumulation in the liver [12], [13]

Prospective cohort studies in Brazil show high rates of reinfection after treatment for geohelminth infection among malnourished children compared to well-nourished children [14]. Moreover, various studies have shown that malnutrition increases susceptibility to infection with soil-transmitted helminthes (STH), which compromise the nutritional status of the host [6], [8], [9], [15]–[19]. Hookworm infection has been associated with a specific malabsorption syndrome that is correlated in severity with the parasitic load [20], [21]. Chronic protein loss during severe hookworm infection can result in hypoproteinemia and anasarca, which is characterized by excessive generalized liquid in the interstitium and inside cells and causes many clinical symptoms [22]. In human infections, the loss of iron in the form of hemoglobin tends to be more critical than protein loss. As stated earlier, severe iron-deficiency anemia is the classic symptom of massive hookworm infection. In areas where children's diets are not sufficient, these losses become severe and may lead to kwashiorkor [8].

Most cases of ancylostomiasis do not result in severe anemia; however, persistent infections and reinfections do result in malnourishment, thereby compromising the cognitive and physical development of children [23] Furthermore, malnourished people are more susceptible to infection; in this way, these factors form a vicious cycle. Work analyzing the association between nutritional deficiency and helminth infection among children from the Philippines has shown a close association between the number of children harboring hookworms and other helminthes and a low intake of nutrients, protein [24].

Protein deficiency consistently interferes with resistance to infections because most immune responses depend on cell replication and the production of active protein compounds that cannot be synthesized without an appropriate balance of amino acids [19], [25]. Studies of hookworm infection and malnutrition in humans are hampered by the fact that malnutrition has many causes. In fact, no study using experimental models of malnutrition to evaluate the association between malnutrition and hookworm infection has been performed to date. Therefore, in this study, a malnutrition model was established in the hamster, an established model of *A. ceylanicum* infection, to assess the effects of low protein intake on the course of *A. ceylanicum* infection and concomitantly analyze how infection affects the nutritional status of the host. Using this model, we evaluated the hypothesis that pathogenesis due to helminth infection is exacerbated by host protein deficiency.

## Materials and Methods

### Ethics statement

All animal procedures were approved by the animal-care ethics committee of the Federal University of Minas Gerais – UFMG (Protocols # 0666/2008 and 194/2011) and were performed under the guidelines from CONCEA - CONSELHO NACIONAL DE CONTROLE DE EXPERIMENTAÇÃO ANIMAL (Brazilian Council of Animal Experimentation) and strictly followed the Brazilian law for “Procedures for the Scientific Use of Animals” (11.794/2008).

### Experimental design

Female hamsters have been routinely used as models for *A. ceylanicum* infection because they are less aggressive than males. Because older animals present a lower infection rate, 4- to 6-week-old hamsters (*Mesocricetus auratus*), were selected for this study. The animals are housed in the Animal Facility in the Department of Parasitology, UFMG, which is not a germ-free system. Therefore, to ensure that the animals were free of helminth infection, they were treated with one dose 5 mg/kg Ivomec (Ivomec Gold 1% - Merial saúde animal Ltda, SP, Brazil) via gavage 30 days prior to *A. ceylanicum* infection. All animals were fed *ad libitum* with manipulated diets as described in **Table 1** [26], for 4 weeks before infection and throughout the experimental period. The hamsters' water and food consumption and weight were measured weekly.

**Table 1:** Composition of diets prepared for the control and malnourished hamsters.

Composition	Normal diet (g/kg)	Hypoproteic diet (g/kg)
Casein	245	60
Metionin	5	0
Vegetable oil	200	200
Corn starch	489.992	679.992
Mix of vitamins^a^	15	15
Mix of minerals^a^	35	35
Cellulose	10	10
BHT	0.008	0.008
Water	qsp^b^	qsp^b^
**Kcal**	**4760**	**4760**
**% protein**	**25**	**6**

**modified from DiBattista** [26] **.**

a acquired from Rhoster Indústria e Comércio Ltda (São Paulo, Brazil).

b amount sufficient to form a pellet feed.

**Figure 1:**
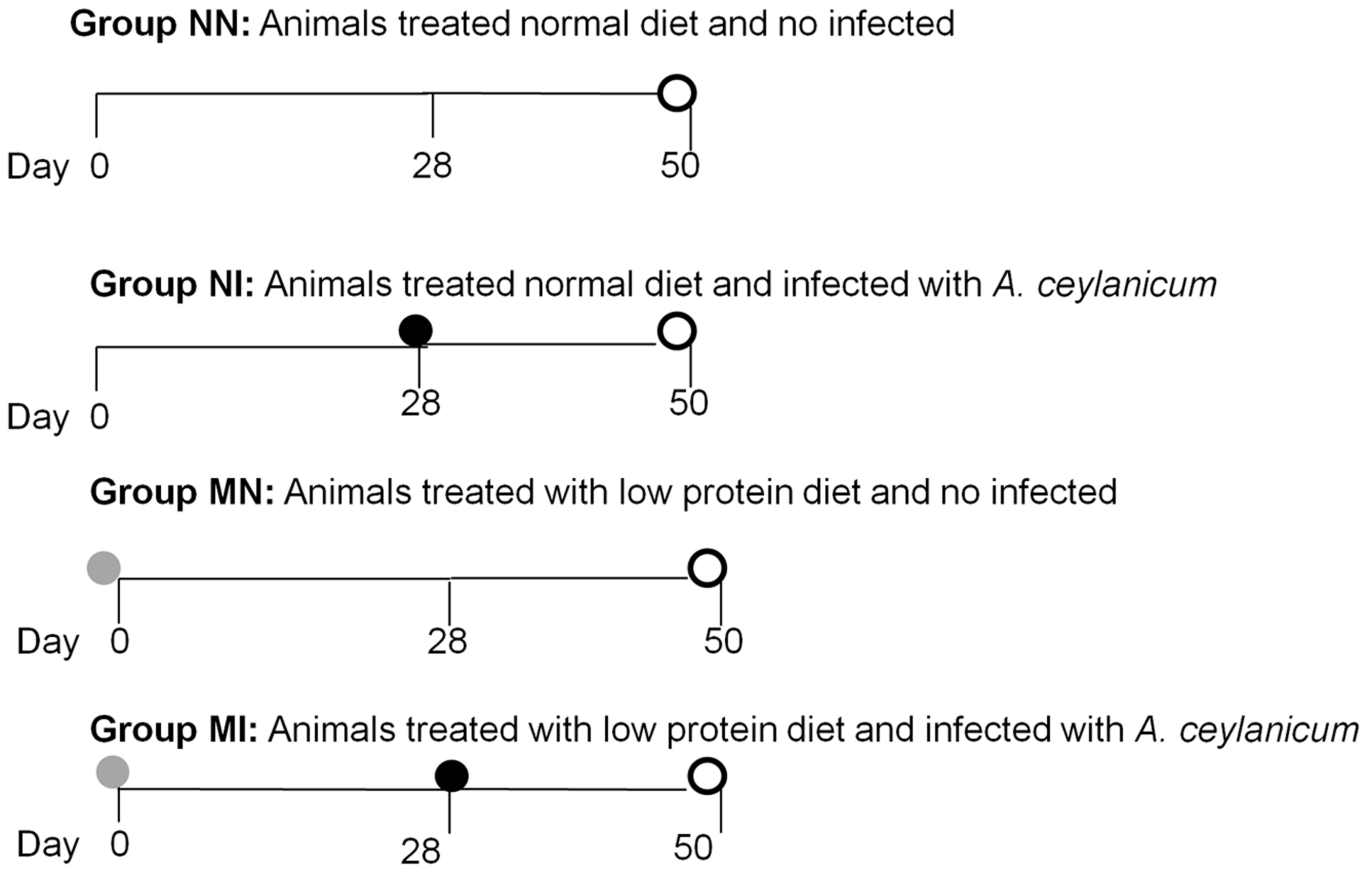
Experimental design. Vertical bars indicates blood collection; Grey circle indicates start of the low protein diet; Dark circle indicates *A. ceylanicum* inoculation and Open circle indicates animal's euthanasia.

Animals were randomly divided into four groups of ten animals each according to diet (normal or hypoproteic) and infection status: i) fed a normal diet (normonourished) and non-infected (NN); ii) fed a normal diet (normonourished) and infected with 50 third-stage larvae – L3 of *A. ceylanicum* (NI); iii) fed a hypoproteic diet (malnourished) and non-infected (MN); or iv) fed a hypoproteic diet (malnourished) and infected with 50 third-stage larvae – L3 of *A. ceylanicum* (MI). The experimental design is shown in Figure 1.

Hamsters were orally inoculated with *A. ceylanicum* via gavage into the upper digestive tract. Thirteen days after inoculation (DAI), feces were collected directly from cages every two days to determine the number of eggs using a McMaster chamber [27]. Individual determination of eggs per gram of feces (EPG) was not performed due to the small volume of stool obtained from each animal. To prevent the spontaneous elimination of adult worms, at 22 DAI, the animals were fasted for 12 h and then killed using an overdose of anesthetic solution (45 mg/kg xylazine cloridrate plus 240 mg/kg ketamine – Xilazin and Cetamin, Syntec, Brazil, administered intraperitoneally).

### Worm recovery

The small intestine was removed and opened in a Petri dish containing PBS, and adult parasites were recovered from the intestinal mucosa. The worms were counted, sexed and separated (fresh or frozen at −20°C) for subsequent antigen preparation.

### Blood collection and hematological parameters

On the first day of the diet (day 0), the day of infection (day 28) and the day of sacrifice (day 50), the animals were fasted for 12 h prior to blood collection. Five hundred microliters (0.5 mL) of blood was individually collected from the retro-orbital plexus [28]. One hundred microliters of blood was used to measure the fast glycemic index, and the remaining material was used to perform a complete blood count (Abacus Junior Vet, Diatron, Austria) and to obtain plasma to assess the cellular response. Reference values for hamsters were obtained from Mitruka and Rawnsley apud Gad [29].

### Visceral adiposity and lean body mass index

To determine the index of visceral adiposity, visceral adipose tissue recovered from each animal after euthanasia was weighed, and the value was corrected for their respective body weights. The lean body-mass index was calculated from the amount of visceral adipose tissue in grams subtracted from the total weight of the animal before euthanasia.

### Biochemical parameters

The following biochemical parameters were evaluated from animal's serum: fasting glucose, total protein, total cholesterol, albumin, HDL cholesterol and triglycerides. Cholesterol, triglycerides and total protein were measured from liver tissue, and cecum contents. All measurements were performed using commercial kits (Doles, Goiânia, Brazil) according to the manufacturer's recommendations. The total lipid contents of the liver, muscle and from cecal feces were measured as previously described by Folch et al. [30]

### Adult crude and excretion-secretion (ES) antigens preparation

Fresh axenic adult worms were washed extensively in sterile PBS and added to 15-mL plastic tubes containing RPMI 1640 culture medium and a solution of antibiotic/antimycotic 6% p/v (Invitrogen, USA) at a concentration of 15 adult worms per milliliter of medium. Cultures were incubated for 18 h at 37°C under 5% CO_2_. The culture medium was centrifuged (15,550× *g* for 3 min at 4°C), and the supernatant containing the ES products was concentrated using a Vivaspin 20 5 kDa MWCO tube (GE Healthcare, USA). Sediment containing adults was washed in sterile PBS, and crude antigen was obtained by mechanically macerating the adult worms in a tissue grinder (Tissue Grinder, Fisher Scientific, USA). The extract was centrifuged to separate debris (14,000 rpm for 10 min at 4°C). The protein concentration was measured using a BCA Protein Assay kit (Thermo Scientific, Pierce, USA), as recommended by the manufacturer. The supernatant aliquots were stored at −80°C until analysis.

### Immunological parameters

The humoral response against adult *A. ceylanicum* crude and ES antigens was evaluated by measuring the total anti-hookworm IgG level in plasma by ELISA. Briefly, plates (BD Falcon, USA) were coated with 1 µg/mL of crude antigen or 5 µg/mL of ES antigen in carbonate buffer (0.05 M NaHCO_3_, pH 9.6) for 18 h at 4°C. The plate was blocked for 90 min at 25°C in a PBS-0.05% Tween 20 (PBST) solution containing 3% casein (Molico - Nestlé). Plasma from each hamster was analyzed individually (1∶100) and incubated for 18 h at 4°C. The secondary antibody (Biotinylated anti-Armenian and Syrian hamster IgG - BD Pharmigen), diluted 1∶5000, was incubated for 2 h at 4°C. Streptavidin (Sigma Aldrich, USA), diluted 1∶3000 in 0.05% PBST, was then added, and the plate was incubated for 20 min. The assay was developed using TMB (BD OptEIA, USA) for 10 min in the dark. The reaction was stopped by adding 100 µl of 2 M H_2_SO_4_ to each well of the plate. The plate was read at 450 nm on a spectrophotometer (VersaMax Microplate Reader, Molecular Devides Inc., USA) using the SoftMax Pro Software. After each incubation step (except after the incubation with TMB), the plate was washed five times with 0.05% PBST.

### Statistical analysis

The results were expressed as the arithmetic mean (black line) ± standard deviation (SD). The Grubbs test was used to detect outliers, which were removed from the analysis. The effects for each treatment/infection were tested using ANOVA and Tukey's test (parametric data) or Kruskal-Wallis test with Dunn's post test (non-parametric data), with a 5% level of significance (p<0.05) using the statistical package Graph Pad Prism 5.0.

## Results

### Malnutrition establishment

**Figure 2:**
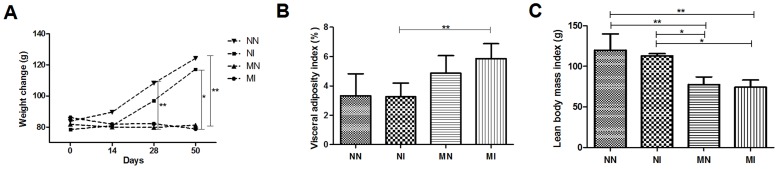
(A) Weight change in grams of the average weight of the groups of hamsters subjected to control or low-protein diet during the experiment (over 50 days); (B) Visceral adiposity index (g) after 50 days of experiment; (C) Lean body mass index (g) after 50 days of experiment. Negative control group (NN), control infected with A. ceylanicum (NI), malnourished negative (MN) and malnourished infected with *A. ceylanicum* (MI). n = 5/6/10/7 hamsters per group, respectively. * = P<0.05, ** = P<0.01.

In all groups, regardless of diet or infection status, some animals died before the end of the study. Hamsters are very sensitive and may have been stressed by the change in diet because even the complete diet was manipulated to avoid major differences in consistency compared to the deficient diet. Therefore, the experiments ended with 5, 6, 10 and 7 animals in groups NN, NI, MN and MI, respectively. Malnutrition was confirmed by the change in the animal's weight and the lean body-mass index (**Figure 2**). Differences in weight were observed mostly between the control groups (normal diet - N) and the groups fed a low-protein diet (malnourished - M), with more weight gain observed in the control animals (NN and NI) compared to malnourished animals (MN and MI; Figure 1A). The average weight of all animals at the beginning of the experiment was 82.67+/−7.60 g. After 28 days of the specific diets, the average weights of the groups were 102.60+/−13.26 g in the normal-diet group and 80.71+/−8.64 g, (p<0.001) in the malnourished group.

At the end of the experiment (22 days post-infection), the average weights for each group were 124.20+/−22.14 g in the negative control group (NN), 117.20+/−2.95 g in the control infected (NI) group,) 81 30+/−9.80 g in the malnourished negative (MN) group and 78.86+/−9.62 g in the malnourished infected (MI) group. The final weights after infection showed little variation within each group, indicating that the weight loss was due to the host nutritional state and was independent of infection (Figure 2A). Visceral adiposity (in grams) was higher in the malnourished groups (p<0.01) compared to the normal groups, indicating that the higher amount of carbohydrate in the diet resulted in an increased gain of visceral fat (Figure 2B); however, the low amount of protein resulted in a loss of lean body mass in the MI group compared to the NI group (p<0.05; Figure 2C).

### Parasitological parameters

**Figure 3:**
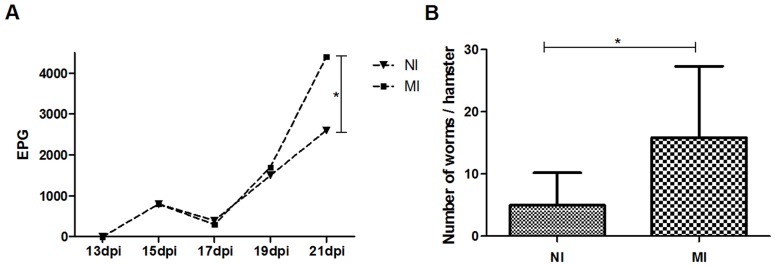
(A) Elimination of *A. ceylanicum* eggs of per gram of feces (EPG) from the 13th DAI (days post infection) until 21 DAI. n = 6/7 hamsters per group, respectively. * = P<0.05. (B) Number of adult *A. ceylanicum* worms recovered from the small intestine of each hamster at 22 DAI. Control groups infected (NI) and malnourished infected (MI).

The number of eggs per gram of feces 21 DAI was significantly higher in the MI group compared to the NI group (P<0.005; Figure 3A). The number of adults worms recovered from each animal from the MI group was significantly higher compared to the control infected group (NI; p<0.005; Figure 3B); however, although the number of eggs was higher in the MI group, the fecundity rate was 42% higher in the NI group compared to the MI group. The oviposition rate of females in the NI group was 144 eggs/female, and that of the MI group was 84 EPG/female (data not shown), showing that the MI group has a lower fecundity rate compared to the control group.

### Hematological analysis

**Figure 4:**
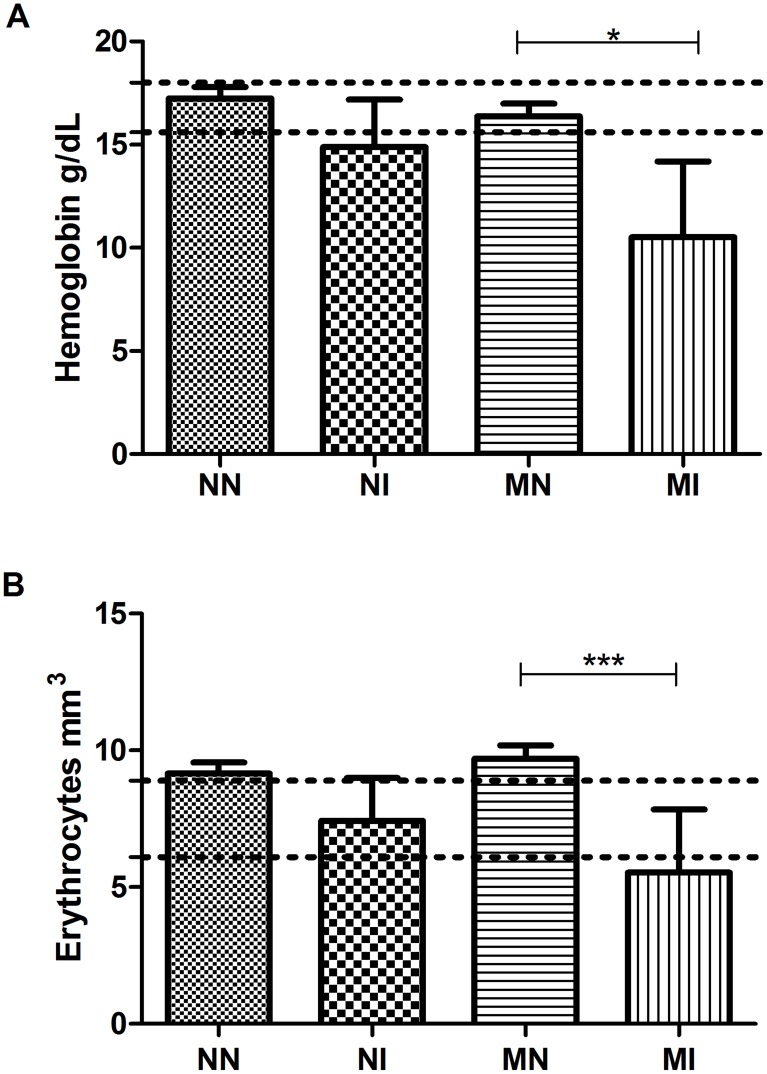
CBC of hamsters performed on day 50 of the experiment. (A) Hemoglobin levels are listed in grams per deciliter; (B) Global count of erythrocytes per cubic millimeter. Negative control group (NN), control infected with *A. ceylanicum* (NI), malnourished negative (MN) and malnourished infected with *A. ceylanicum* (MI). Dotted line: physiological values for hamster according Mitruka and Rawnsley (1981). n = 5/6/10/7 hamsters per group, respectively. * = P<0.05, *** = P<0.001.

On day 50 of the experiment, a decrease in the hemoglobin levels of the MI group was observed compared to the MN (p<0.05) and NI groups, demonstrating that malnutrition exacerbated the pathogenesis caused by infection (Figure 4A). Similarly, after 50 days, a decrease of erythrocyte number was observed in the MI group compared to the MN (p<0.001) and NI groups, indicating that the infection resulted in the decrease of erythrocytes exacerbated by the malnutrition state (Figure 4B). The total leukocyte count showed no significant difference between the experimental groups (data not shown). Similarly, the number of lymphocytes, monocytes and circulating neutrophils showed no significant difference between experimental groups (data not shown).

### Humoral immune response

**Figure 5:**
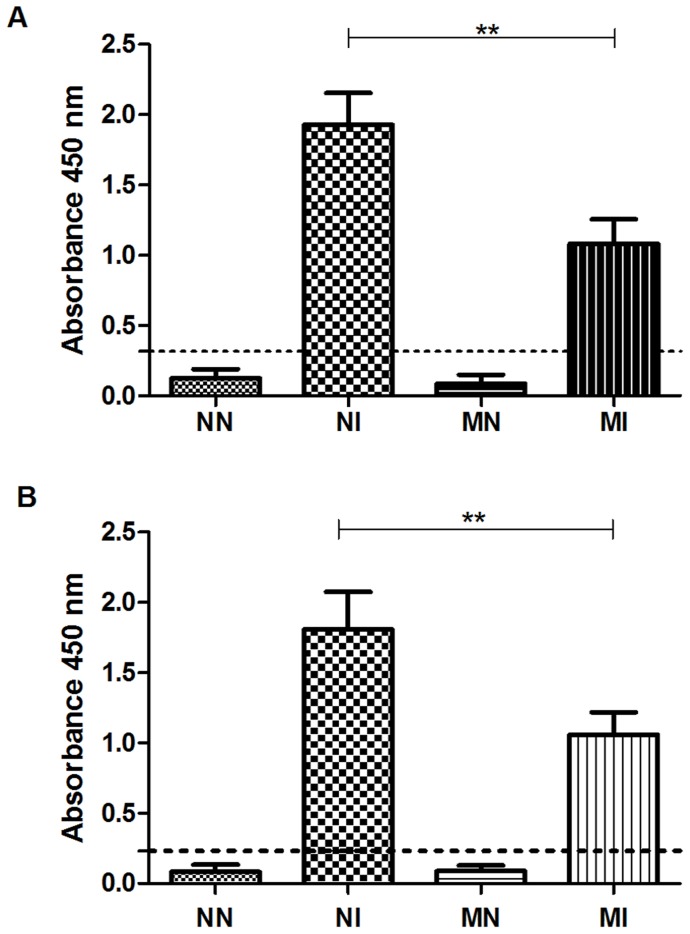
Levels of total IgG in plasma of hamsters antigens were: (A) crude extract of *A. ceylanicum*; (B) ES products. Negative control group (NN), control infected with *A. ceylanicum* (NI), malnourished negative (MN) and malnourished infected with *A. ceylanicum* (MI). Dotted line: cut-off. Plasma obtained at 22 DAI. n = 5/6/10/7 hamsters per group, respectively. ** = P<0.01.

Figure 5 shows that the NI group had a greater immune response than did the MI group against both antigens tested, i.e., crude extract and ES products (p<0.01), suggesting that the low amount of protein in the diet accounted for the decreased production of IgG.

### Biochemical parameters in plasma

**Figure 6:**
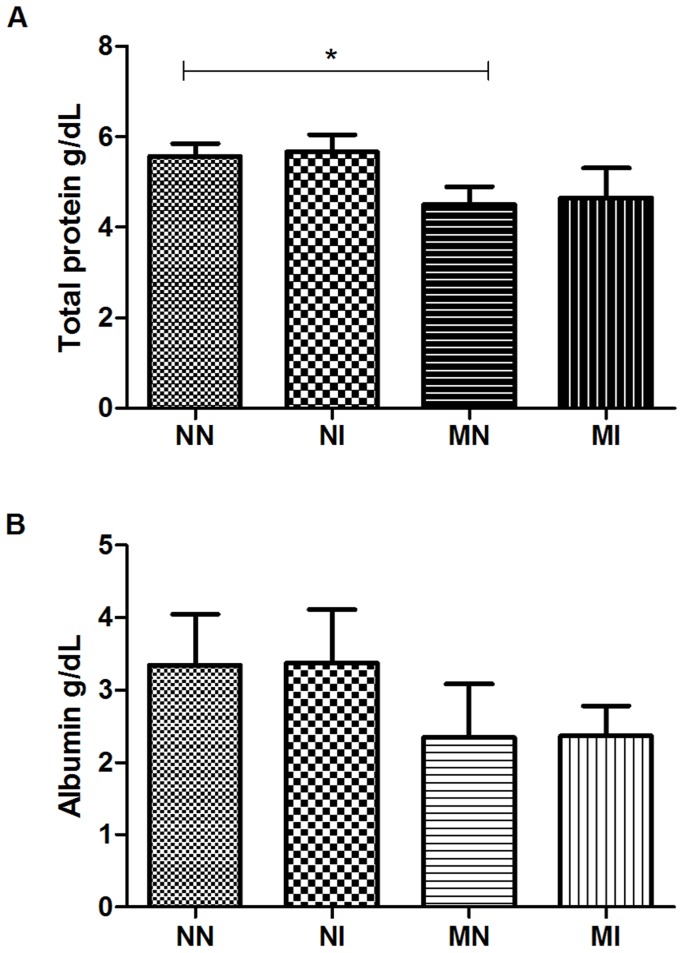
Biochemical parameters in plasma of hamsters on day 50 of the experiment. (A) Total protein in grams per deciliter; (B) Albumin gram per deciliter. Negative control group (NN), control infected with *A. ceylanicum* (NI), malnourished negative (MN) and malnourished infected with A. *ceylanicum* (MI). n = 5/6/10/7 hamsters per group, respectively. * = P<0.05.

A significant decrease in the amount of total protein was observed in the blood of the MN group relative to the NN group (p<0.05), showing that a protein-deficient diet plays a role in the decrease in plasma proteins (Figure 6A). The levels of glucose (data not shown) and albumin (Figure 6B) showed no significant differences among the groups, showing that neither malnutrition nor infection influenced these parameters.

### Evaluation of lipid levels

**Figure 7:**
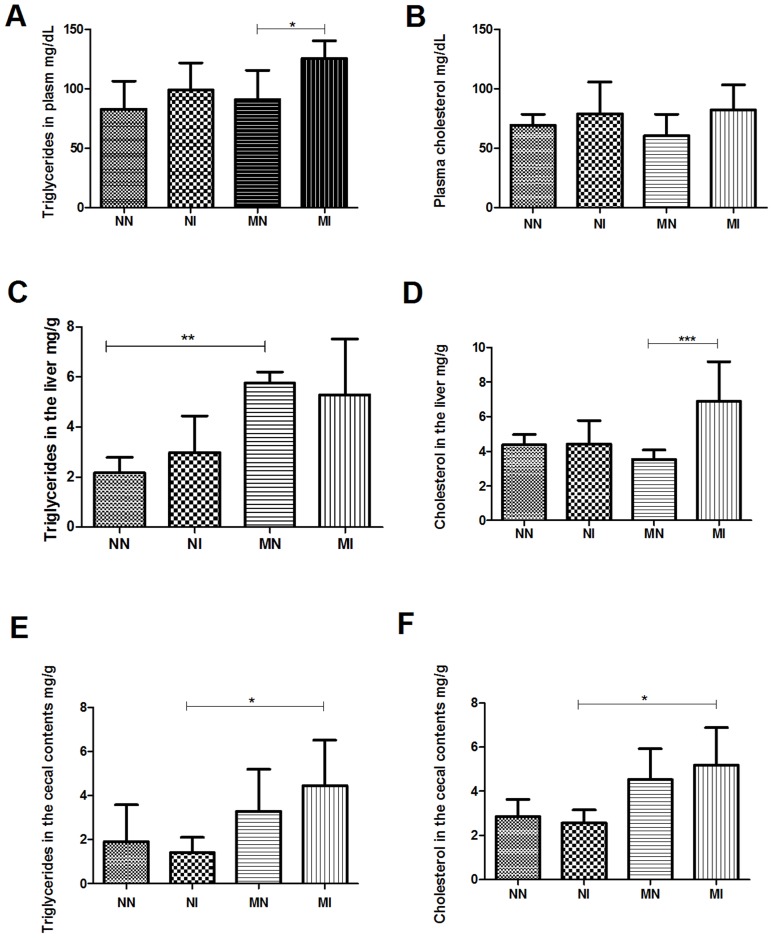
Lipid content on day 50 of the experiment. (A) Triglycerides in plasma in milligrams per deciliter; (B) Cholesterol in plasma gram per deciliter (C) Triglycerides in the liver milligram per gram; (D) Cholesterol in the liver in milligrams per gram; (E) Triglyceride in cecal contents milligram per gram, (F) Cholesterol in cecal contents in milligrams per gram. Negative control group (NN), control infected with *A. ceylanicum* (NI), malnourished negative (MN) and malnourished infected with *A. ceylanicum* (MI). n = 5/6/10/7 hamsters per group, respectively. * = P<0.05, ** = P<0.01, *** = P<0.001.

Triglyceride analysis on day 50 of the experiment showed a significant increase in plasma triglycerides in the MI group compared to the MN group (p<0.01; Figure 7A). The analysis of total plasma cholesterol showed no statistically significant difference among the groups (Figure 7B). In liver tissue, the MN group showed a significant increase in the amount of triglycerides relative to the NN group (p<0.01), while the malnourished, infected animals showed no significant difference compared to the other groups (Figure 7C); however, the MI group showed a significant increase in cholesterol levels in relation to the MN group (p<0.001), demonstrating the effect of infection on the deposition of cholesterol in the liver of malnourished animals (Figure 7D). The analysis of cecal lipids showed an increase in the amounts of triglycerides (Figure 7E) and cholesterol (Figure 7F) excreted in feces in the MI group compared to the NI group (p<0.05) in both cases.

## Discussion

Clinical manifestations of protein deficiency include weakness, cachexia, growth retardation, muscle wasting, steatosis, dermatosis, decreased immune response, increased susceptibility to invasion by pathogens and general debility. Furthermore, general anatomical changes, such as tissue hypoplasia and atrophy, are generally observed and can consequently affect physical and cognitive development [12], [31]. In this context, this study sought to evaluate how a low-protein diet influences the course of infection by *A. ceylanicum*. To achieve this goal, nutritional parameters, particularly the weight gain or loss of the animals according to their specific diets, were evaluated.

The increased visceral adiposity index in malnourished animals, especially in the MI group, may have occurred because of the higher concentration of carbohydrates in the low-protein diet compared to the control diet. If they are not converted into energy or other metabolites, excess carbohydrates are converted into fatty acids that are incorporated into triglycerides, which can be stored as fat in adipose tissue [32]. Similar results were observed in experiments in mice, in which a higher percentage of body fat was observed in animals fed with a low-protein diet compared to mice fed with a high-protein diet [33]. Additionally, a decrease in lean body mass was observed in the malnourished animals compared to the control group. These data corroborate studies showing that muscle tissue from animals fed protein-deficient diets is degraded to provide amino acids for the synthesis of more important proteins in the liver [34]

Host susceptibility to parasitism was measured by analyzing the parasite load and eggs eliminated during infection. The low-protein diet led to a decreased host response to the infection, favoring the establishment of a greater number of worms in the intestine compared to the control infected group. Although more parasites were recovered from the MI group, the fertility rate of females from this group was 58% lower compared to those from the NI group. This difference may be the result of the impaired nutritional status of the host in the malnourished group. The scarcity of nutrients and intraspecific competition by the parasite may limit the resources required for the development of adult parasites and thus for egg production. For gastrointestinal parasites, the relationship between population density and the release of eggs in the feces is usually inversely proportional [35], [36]. Other studies have demonstrated a reduced production of eggs by gastrointestinal parasites in animals fed low-protein diets [37], [38]. A study using sheep infected with *H. contortus* and fed a low-protein diet showed that these animals presented more severe clinical signs than did sheep maintained with an adequate-protein diet; however, the fecal egg counts, oviposition rate and number of adult worms recovered were similar in both groups [39]

In response to infection, host defense involves many events that consume additional anabolic energy; consequently, the nutritional status of the host is critical to the outcome of infection. Almost all dietary nutrients play a crucial role in maintaining an optimal immune response, so deficient or excessive nutrient intake can profoundly affect immune status and susceptibility to a variety of pathogens [40], [41]. During hookworm infection, the development of a Th2 response coincides with the patency of infection and occurs synchronously with the modulation of the Th1 response [42], [43]. In this study, animals from the MI group showed a significantly lower immune response compared to those from the NI group. This difference may have been caused by a low level of amino acids, which would affect the formation of antibodies [44], [45] and is correlated with lower plasma-immunoglobulin concentration [46]. In *Leishmania chagasi* vaccination studies using BALB/c mice fed either a control diet or a diet low in protein, iron and zinc, the vaccine efficiency was higher in the mice fed a control diet. In animals fed the deficient diet, the immune response after vaccination was much lower, and the number of parasites found in the liver and spleen was much higher compared to the control group [39]. Another study showed that sheep that are resistant or susceptible to the parasite *H. contortus* and fed a normal diet showed higher IgA activity compared to ewes fed a diet deficient in protein [25].

In this study, we observed a decrease in the amount of total serum protein in malnourished animals compared to control animals, showing that the low-protein diet was a determining factor for the decrease in plasma proteins; however, there were no significant differences in albumin levels among the experimental groups. Data from the literature show that serum albumin has a tendency to gradually decrease according to the severity of anemia [47], and hypoalbuminemia has been observed in mass hookworm infection [48]. The lack of a decrease in this parameter may be the result of the low inoculum and/or to the short period of exposure to infection. Serum-albumin levels respond slowly to malnutrition because this protein has a relatively long half-life in the organism (14–20 days) and a large reservoir (4–5 g/kg of body weight), making it a poor indicator of early protein depletion [49]. In fact, approximately 60% of the total protein content of the body is found outside the bloodstream. When the serum-albumin concentration decreases at the onset of malnutrition, extravascular albumin moves into the blood and helps maintain the normal serum concentration despite the protein and energy deficits, so the serum albumin concentration does not decrease in cases of light or moderate malnutrition [50]. A study of malnutrition and infection with *Heligmosomoides polygyrus* showed that the albumin levels in malnourished animals did not change relative to control animals, but the immune response was affected by the low-protein diet, showing that homeostatic mechanisms enabled the host to maintain plasma-albumin concentration at the expense of host immune function [51]. Except for the total plasma protein, which decreased in the MN group in comparison to the NN group, no alterations in hematological parameters were observed in the malnourished animals compared to the control-diet animals. Nevertheless, a decline in hemoglobin and erythrocytes was observed in the MI group compared to the MN group, demonstrating that malnutrition exacerbated the pathogenesis caused by infection.

Infection associated with malnutrition worsens dyslipidemia, causing an increase in plasma triglycerides. Although no differences were observed in the cholesterol in the blood, liver cholesterol data were different among the groups. In this organ, the MI group showed higher cholesterol levels than did the MN group, showing that infection caused fat deposition in the liver of these animals during malnutrition. The concentration of triglycerides in the liver was higher in the MN group than in the NN group, showing that malnutrition favored the accumulation of triglycerides in the liver. In general, increasing values of total lipids were observed in malnourished animals in comparison to control animals. These data show that infected and protein malnourished animals were more likely to develop hepatic steatosis, which occurs in children eating a low-protein diet [12]. The increased lipid concentration in the liver and blood is related to the conversion of the excess carbohydrate into fatty acids that will increase the concentration of triglycerides in the liver. The increase in plasma triglycerides suggests that the excess lipids are being transported to adipose tissue, as reflected by the increased adiposity in both malnourished groups.

Protein deficiency may be an important factor in the pathogenesis of fat malabsorption [48]. Some studies have shown abnormal nutrient absorption and increases in fecal fat excretion associated with severe hookworm infections, particularly when associated with a deficit of protein in the diet [20]; [48]. Here, we observed differences in the amounts of triglycerides and cholesterol excreted in the stool, which were higher for the MI group than it was for the NI group. These results indicate that infection associated with malnutrition caused a greater loss of these molecules in these animals, confirming the hypothesis that both factors are responsible for the malabsorption syndrome. The confirmation of the pathological association of malnutrition and infection does not explain the molecular mechanisms involved. Experiments evaluating the role of molecules secreted/excreted by the parasite in the intestine of the host could further clarify these mechanisms.

In this study, established a protein-malnutrition model in the hamster with the potential to improve studies of malnutrition and parasite infection. Furthermore, this work supports the hypothesis that the co-occurrence of malnutrition and hookworm infection has exacerbates both conditions. This interaction constitutes a major public-health problem: hookworms occur largely in poor areas where are many children have protein-deficient diets and the prevalence of other STH diseases is also high.
